# The cancer survival index—A prognostic score integrating psychosocial and biological factors in patients diagnosed with cancer or haematologic malignancies

**DOI:** 10.1002/cam4.4697

**Published:** 2022-03-22

**Authors:** Alexander Gaiger, Simone Lubowitzki, Katharina Krammer, Elisabeth L. Zeilinger, Andras Acel, Olivera Cenic, Andrea Schrott, Matthias Unseld, Anahita Paula Rassoulian, Cathrin Skrabs, Peter Valent, Heinz Gisslinger, Christine Marosi, Matthias Preusser, Gerald Prager, Gabriela Kornek, Robert Pirker, Günther G. Steger, Rupert Bartsch, Markus Raderer, Ingrid Simonitsch‐Klupp, Renate Thalhammer, Christoph Zielinski, Ulrich Jäger

**Affiliations:** ^1^ Department of Medicine I, Division of Haematology and Haemostaseology Medical University of Vienna Vienna Austria; ^2^ Department of Medicine I, Division of Oncology Medical University of Vienna, Comprehensive Cancer Centre, Medical University Vienna – General Hospital Vienna Austria; ^3^ Statistikambulanz KG Leobendorf Austria; ^4^ Department of Medicine I, Division of Palliative Medicine Medical University of Vienna Vienna Austria; ^5^ Ludwig Boltzmann Institute for Haematology and Oncology Medical University of Vienna Vienna Austria; ^6^ Clinical Institute of Pathology Medical University of Vienna Vienna Austria; ^7^ Department of Laboratory Medicine Medical University of Vienna Vienna Austria

**Keywords:** prognosis, prognostic factor, psychosocial studies, survival

## Abstract

**Objective:**

We aimed to investigate whether (1) psychological and social indicators influence survival in patients diagnosed with cancer or haematologic malignancies when important biological aspects are controlled for, (2) psychological, social and biological indicators can be utilised to design one collated index for survival, usable in clinical practice to identify patients at risk of shorter survival and to improve personalised healthcare provision.

**Methods:**

In this cross‐sectional study, 2263 patients with cancer or haematologic malignancies participated. We analysed 15 biological, psychological and social indicators as risk factors for survival with a Cox proportional hazards model. Indicators significantly associated with survival were combined to compute models for the identification of patient groups with different risks of death. The training sample contained 1122 patients. Validation samples included the remaining 1141 patients, the total sample, as well as groups with different cancer entities.

**Results:**

Five indicators were found to significantly impact survival: Cancer site (HR: 3.56), metastatic disease (HR: 1.88), symptoms of depression (HR: 1.34), female sex (HR: 0.73) and anaemia (HR: 0.48). Combining these indicators to a model, we developed the Cancer Survival Index, identifying three distinct groups of patients with estimated survival times of 47.2 months, 141 months and 198.2 months (*p* < 0.001). Post hoc analysis of the influence of depression on survival showed a mediating effect of the following four factors, related to both depression and survival: previous psychiatric conditions, employment status, metastatic disease and haemoglobin levels.

**Conclusions:**

Psychosocial and biological factors impact survival in various malignancies and can be utilised jointly to compute an index for estimating the survival of each patient individually—the Cancer Survival Index.

## Graphical abstract

We analysed 15 biological, psychological and social indicators as risk factors for survival in 2263 cancer patients with a Cox proportional hazards model. Five indicators, that is cancer site, metastatic disease, depression, female sex and anaemia, were significantly associated with survival and combined to compute the Cancer Survival Index.

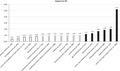

## 1 BACKGROUND

Cancer is one of the leading causes of death. Cancer incidence is rising worldwide, due in part to the growing and ageing global population. Statistically, overall survival (OS) of cancer patients is constantly improving.^1^ On an individual level, however, survival varies tremendously. There is solid evidence regarding the impact of biological factors on OS in cancer patients. These classical factors include tumour biology, microenvironment and host‐related (clinical) factors. In addition to established tumour site‐specific scores or the TNM (tumour‐node‐metastasis) staging system, factors impacting OS of cancer patients include sex, age, cancer site or anaemia.^2^, ^3^, ^4^, ^5^

Indicators of socioeconomic status, including household income, education level, and social support,^6^, ^7^, ^8^, ^9^, ^10^, ^11^, ^12^, ^13^ as well as psychological factors, such as general psychological distress, anxiety, and depression,^14^, ^15^, ^16^, ^17^, ^18^, ^19^, ^20^, ^21^, ^22^ have been recognised as important to OS in cancer patients, but are usually not considered in risk assessment, clinical trial design and patient management. Existing prognostic scores for cancer survival mostly focus on biological aspects, but do not include psychosocial or socioeconomic factors.^23^, ^24^ However, we believe that there is a medical need to do so:

1. Not considering psychosocial variables in study design can bias the results of clinical trials. If groups differ in important psychosocial indicators, the apparent association between the respective factor of interest and the outcome can be flawed.
2. Research shows that psychosocial indicators influence adherence to screening and compliance with cancer treatment, are related to comorbidities, and ultimately impact survival.^6^, ^7^, ^25^, ^26^
3. Growing financial constraints^27^ and legal aspects (informed consent may be impaired by e.g., post‐traumatic stress disorder, chronic fatigue, depression etc.)^28^ provide further stimuli to evaluate the benefits of a biopsychosocial model in clinical oncology.
4. Incorporating psychosocial factors into clinical oncology supports the aim of personalised medicine, which is to implement the most appropriate treatment and healthcare strategy for each patient based on their respective genetic and environmental characteristics.^29^

In the present study we aimed to investigate two aspects: First, the influence of psychosocial indicators on OS in cancer patients when important biological aspects are considered simultaneously. Second, whether and how psychosocial and biological indicators can be utilised to design one collated index for survival. Such an integrated approach could improve assessment of patient survival, patient management as well as clinical study design.

## 2 METHODS

### 2.1 Patients

In total, 2263 patients with various malignancies (1097 men and 1166 women) were included. All patients gave written informed consent and were tested between March 2006 and November 2010 at the Department of Medicine I with its Clinical Divisions of Haematology and Oncology of the Comprehensive Cancer Centre Vienna (www.ccc.ac.at) after approval by the Ethical Committee of the Medical University of Vienna (EC number: 473/2006). Patients’ characteristics are shown in Table S1.

### 2.2 Measurements

Clinical data were obtained from medical records. The Hospital Anxiety and Depression Scale (HADS) was used to assess anxiety and depression. The HADS is a 14‐item self‐administered rating scale, specifically developed for non‐psychiatric medical patients.^30^, ^31^ The HADS was found to be reliable and valid as a screening tool by three comprehensive reviews.^31^ The sociodemographic questionnaire included general data like age, sex, education, employment, income, marital status, number and age of children, previous or present psychiatric conditions. The need for psychosocial support was also recorded.

### 2.3 Selection of risk factors

Fifteen parameters including biological (*k* = 5), psychological (*k* = 4) and sociodemographic variables (*k* = 6) were included in the analysis of OS among 2263 patients diagnosed with various malignancies.

Biological (somatic) indicators impacting OS were selected based on published data and feasibility.^2^, ^3^, ^4^, ^5^ Based on feasibility we selected psychosocial parameters obtainable by self‐assessment tools. The following factors were included in the analysis: (1) Biological indicators: sex, age, cancer site, presence of distant metastases (M1 vs. M0) and haemoglobin levels (<11 vs. >11 g/dl).^2^, ^3^, ^4^, ^5^ (2) Social indicators: household income, education level, status of employment, marital status, living environment (urban / rural) and children (yes / no).^6^, ^7^, ^8^, ^9^, ^11^ (3) Psychological indicators: previous psychiatric disorders, general psychological distress, and symptoms of anxiety and depression.^15^, ^16^, ^17^, ^18^, ^19^, ^22^

### 2.4 Statistical analysis

The influence of 15 somatic and psychosocial variables on OS was analysed with a Cox proportional hazards model. For identification of independent prognostic factors impacting OS, we used a training sample containing 1122 patients tested within 12 months after diagnosis. The prognostic model was then applied to the validation samples, which consisted of the remaining 1141 patients tested 12 and more months after diagnosis as well as the entire patient cohort. Variables independently associated with OS were identified with multivariate step‐by‐step regression.^32^ The survival index (SI) was validated by using a Kaplan–Meier analysis^33^ to predict time‐dependent survival of different risk groups.

Statistical analyses were performed using SPSS 19.0. In the regression analysis, the cut‐off point for haemoglobin was defined as 11 g/dL based on previous studies. Age was dichotomised using a cut‐off of 60 years. Depression and anxiety were dichotomised based on the established criteria of the HADS score (≥7 for depression and ≥9 for anxiety). Education was dichotomised in low (compulsory/vocational school) and high (secondary school/university degree) education level, the cut‐off being high school graduation, income was grouped below and above 1300 € monthly family net income.

## 3 RESULTS

### 3.1 Analysis of individual features

Mean age of the total sample was 58.1 years (range 18 to 92 years). Mean age of women (*n* = 1166) was 57.5 years (range 18–92 years) and of men (*n* = 1097) 58.6 years (range 18–88 years). The mean duration of follow‐up was 62.6 months (range 1 to 490 months). The OS among all patients at 5 years was 65.7%. Information on 15 factors analysed (somatic: sex, age, cancer site, distant metastases and haemoglobin levels; psychological: previous psychiatric disorders, distress, anxiety and depression; social: household income, education level, status of employment, marital status, living environment and children) was complete for all 2263 patients. As determined by HADS, the prevalence of suspected depression in the sample was 32.9% (HADS cut‐off ≥7), the prevalence of suspected anxiety 24.3% (HADS cut‐off ≥9) (Table S1).

### 3.2 Factors impacting OS in cancer patients in multivariate analysis

**FIGURE 1:**
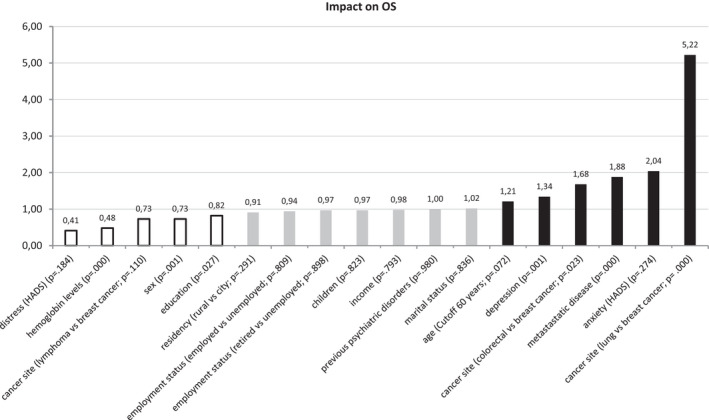
The impact of biological and psychosocial factors on overall survival in 2263 cancer patients. Note. The y‐axis represents the relative risk calculated using multivariate regressions by proportional hazards regression

The impact of the 15 individual factors on OS in multivariate analysis is shown in Figure 1. A step‐by‐step regression analysis identified five characteristics which remained independently significant and predictive of OS in the training as well as the validation samples: cancer site (hazard ratio, HR: 3.56), metastatic disease (HR: 1.92) and depression (HR: 1.42) were related to shorter OS, female sex (HR: 0.75) and haemoglobin levels above 11 (HR: 0.54) correlated with longer OS. (Table 1).

**TABLE 1:** Five factors impact overall survival in cancer patients independent of each other

	*p*	exp(*B*)	Confidence interval	
			Lower value	Higher value
Haemoglobin levels (11 or higher vs. <11)	<0.001	0.54	0.42	0.68
Cancer site (anchor: breast cancer)				
lung & colorectal cancer vs. anchor	<0.001	3.56	2.61	4.86
Metastatic disease (anchor: M1 vs. all other)	<0.001	1.92	1.46	2.53
Sex: female vs. male	0.016	0.74	0.59	0.95
HADS‐depression high	0.001	1.42	1.15	1.76

Abbreviation: HADS, Hospital Anxiety and Depression Scale.

### 3.3 Designing the cancer survival index—CSI

**FIGURE 2:**
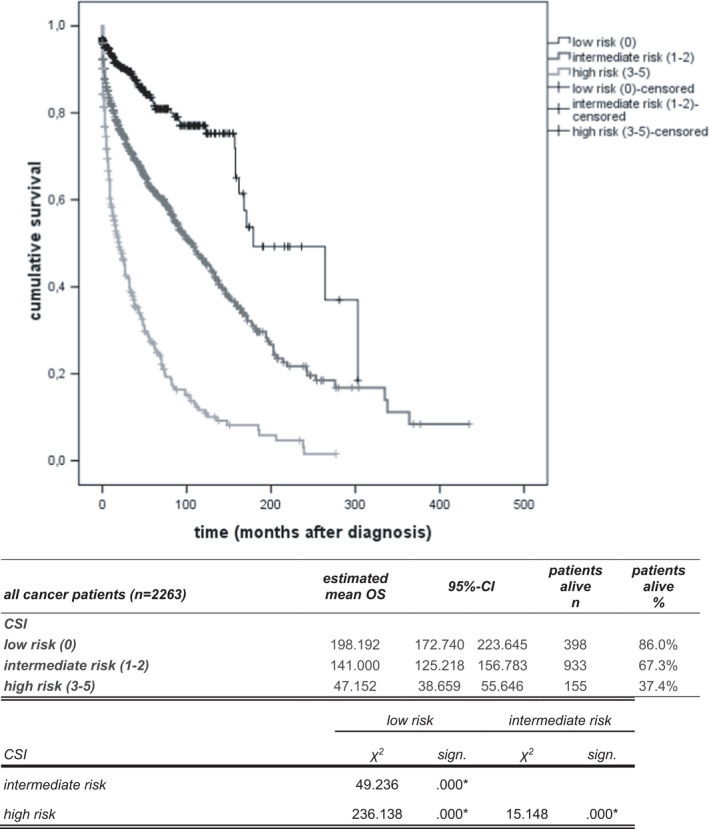
The cancer survival index (CSI) predicts overall survival in 2263 cancer patients

Using these five variables, we designed a model—the cancer survival index (CSI)––for prediction of an individual patient’s risk of death. Patients were assigned to one of the three risk groups based on their number of risk factors: 0, low risk; 1 and 2, intermediate risk, 3–5, high risk(Figure 2). The survival curves and death rates over time for the three risk groups in the all cancer patients sample are shown in Figure 2. Predicted survival time of the three groups were: 198.2 months for the low‐risk group, 141 months for the intermediate‐risk group and 47.2 months for the high‐risk group (*p* < 0.001).

### 3.4 Validation of the CSI in lymphoma, breast, lung, colorectal and ‘other’ cancer patients

Next, we validated the CSI in each of the following cancer entities: lymphoma (*n* = 503), female breast (*n* = 414), lung (*n* = 241), colorectal (*n* = 146) and ‘other’ (*n* = 959) cancer patients. The last group includes various cancer entities that could not be analysed separately due to the small number. Patients were assigned to one of the three risk groups based on their number of risk factors: as lung or colorectal cancer constitute already one risk factor, no patients were included in the low risk group in those two entities. The CSI was predictive for OS in all five cancer subgroups tested, with *p*_*s*_ ≤ 0.003. Predicted survival times are depicted in Table 2.

**TABLE 2:** Predicted survival times in months for different risk groups

Cancer site	Low‐risk group	Intermediate‐risk group	High‐risk group
Lymphoma	233	160	71.4
Breast cancer	223.2	152.1	84
Lung cancer	‐	64.2	20.8
Colorectal cancer	‐	105.4	51.7
Other	135.3	124.9	49.2

### 3.5 Factors associated with depression impact OS

**FIGURE 3:**
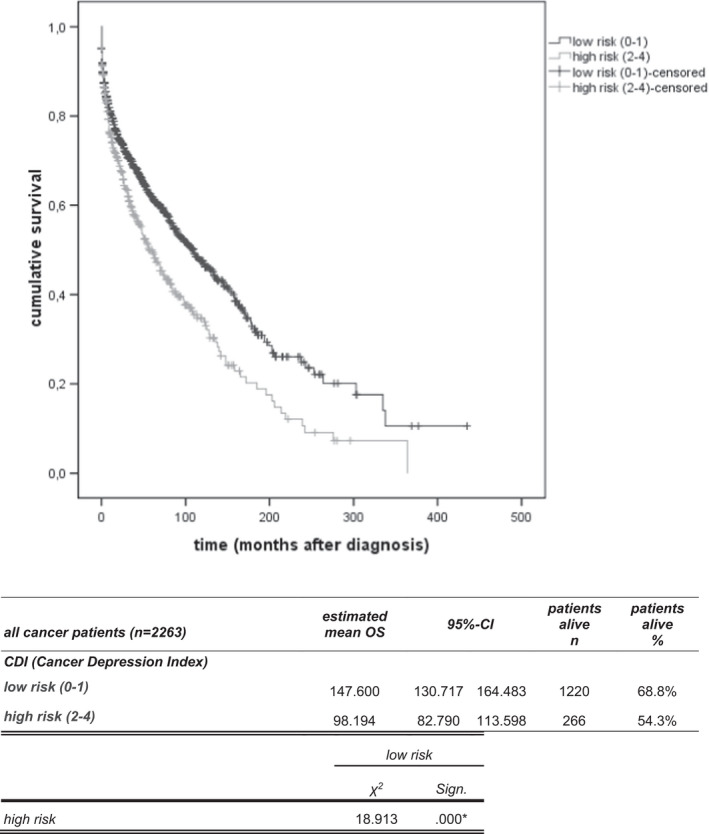
The cancer depression index (CDI) predicts overall survival in 2263 cancer patients

Depression was shown to be significantly associated with OS. To further corroborate the influence of psychosocial factors on OS, we analysed which of the other 14 parameters are associated with depression and developed a model depicting the influence of those factors on survival. After step‐by‐step regression analysis, previous psychiatric disorders (HR 3.43, *p* < 0.001) and presence of distant metastases (HR 1.58, *p* < 0.001) were significantly associated with shorter OS, haemoglobin levels above 11 (HR 0.7, *p* = 0.003) and employment (HR 0.66, *p* < 0.001) were associated with longer OS. Using these variables, we designed a model, the cancer depression index (CDI), to predict an individual patient’s risk of death. Patients were assigned to one of two risk groups based on their number of risk factors: 0 and 1 low risk; 2–4 high risk. The survival curves and death rates over time for the two risk groups in the all cancer patients sample are shown in Figure 3, identifying two groups with predicted survival times of 147.6 (low risk) and 98.2 months (high risk) (*p* < 0.001).

### 3.6 Factors associated with depression impact patient–doctor communication

Correlation analysis indicated that significantly more depressive symptoms were shown by patients with low household income (*p* < 0.001) and low education level (*p* <0.001) (Figures S1 and S2). Education and income significantly affected patient–doctor communication. All patients were offered psychosocial support as part of their treatment programme. Patients with low income or low education—even when depressed—less frequently articulated need for psychosocial support than cancer patients with high income or education (Figures S1 and S2). Patients with high income and education most frequently articulated need for psychosocial support, even when not depressed as documented by low HADS scores. As a consequence, patients with low income and low education received less psychosocial support compared with patients with higher income and education (Figures S1 and S2).

## 4 DISCUSSION

In the present study we combined somatic, psychological and social aspects and established a scoring procedure for estimating the OS in cancer patients. The resulting cancer survival index (CSI) contains aspects easy to asses and is therefore especially usable in clinical practice. Of the 15 factors included in this study, five different parameters are related to OS. Cancer site, metastatic disease, anaemia and sex are well established parameters and their impact on OS is recognised.^2^, ^3^, ^4^, ^5^ Our data demonstrating an effect of depression on OS is in accordance with published reports.^15^, ^16^, ^17^, ^18^, ^19^

Our study results indicate that the influence of depression on OS is mediated by the association of depression with other aspects relevant to survival, including previous psychiatric disorders, employment status, metastatic disease and haemoglobin levels. Combining those factors into one biopsychosocial cancer scoring system is novel and represents the importance of psychosocial aspects—the tumour macroenvironment—for survival.

Data presented in this study demonstrate that patients with low socioeconomic status, measured by household income and education level, are more prone to show symptoms of depression. However, these patients are also less likely to express a need for psychological treatment or psychosocial support, and they are less likely to receive such support. This reflects a highly relevant social problem, as it is related to unequal quality of care based on patients’ social indicators. This disparities in cancer care have been further exacerbated by the recent COVID‐19 pandemic.^34^ Physicians should pay particular attention to possible psychiatric comorbidities and the need for psychosocial support in patients with low socioeconomic status. Also in other studies, need for psychosocial support was an indicator for shorter survival in cancer patients,^35^ and psychosocial interventions were shown to improve patient survival.^36^ Also, low socioeconomic status was found to be associated with an elevated mortality rate in patients with different cancer entities,^13^ as well as in adult and adolescent lymphoma patients.^12^, ^37^ This disparity in survival was partly linked to later diagnosis in patients with low socioeconomic status.^38^ New therapies generally increased survival of lymphoma patients in the last decades. However, in patients with low socioeconomic status this increase in survival is far less than in people with higher socioeconomic status.^39^

Recent publications demonstrate (1) a correlation between lower education and high body mass index in the highly developed and industrialised countries, (2) the impact of high body mass index on all‐cause mortality and (3) that poverty and low education are associated with higher mortality.^6^, ^7^, ^8^, ^9^, ^11^, ^40^ The present data indicate that each of these factors does not act alone but results in a vicious circle aggravating the social impact and burden of cancer and explain how psychosocial factors might complement the predictive value of biological factors identifying patients with a high unmet medical need.

Natural selection describes the process of interplay between somatic and environmental factors resulting in evolution. In a biological concept, selection pressure on the malignant clone might not be restricted to its genetic profile, the tumour microenvironment and immunological factors but might extend to comorbidity, medical infrastructure, patients’ ability to adhere to complex screening and treatment programmes, society’s ability to make such programmes widely accessible etc.—the tumour macroenvironment.

Another hypothesis addressing the ‘mind–body’—interplay includes epigenetic changes. Recent data indicate that psychological and social stressors can induce epigenetic changes.^41^ The impact of epigenetic factors on the survival of cancer patients has been investigated in several research efforts. Further studies need to analyse whether psychosocial factors may lead to epigenetic changes in cancer patients affecting OS.

### 4.1 Study strengths and limitations

The strengths of this study include its large sample size, unicentricity and the availability of simultaneous data regarding somatic, socioeconomic and psychological aspects from each individual. Nonetheless, the following limitations have to be pointed out: First, part of the data used in the present analysis is based on patients’ self‐reports, which may lead to misclassification. Though, research indicates that self‐reported data on cancer and chronic disease can be regarded as reliable.^42^

Second, we used a screening tool, the HADS, to assess depression and anxiety. Screening tools can be over‐inclusive compared to clinical interviews. However, there are studies supporting the reliability and validity of the HADS for detection of anxiety and depression in patients with a somatic illness.^26^, ^31^

Third, the diagnosis of depression was not based on the ‘gold standard’ of clinical interview. However, the HADS is a widely accepted, valid and reliable tool developed to identify anxiety and depression in somatically ill patients.^26^, ^31^ Fourth, our data are derived from a cross‐sectional study. We limited the impact of this fact by validating parameters first in a training set consisting of 1122 patients tested within 12 months after diagnosis, then by applying the model to a validation sample of 1141 patients tested 12 months or more following diagnosis, as well as to the entire cohort of 2263 patients and selected cancer sites.

### 4.2 Clinical implications

Considering the importance of psychosocial factors for the survival of patients with cancer, these aspects should be included in study design of clinical trials as well as in screening and treatment approaches for individual patients. Not considering psychosocial variables in study design can bias the results of clinical trials. If groups differ in important psychosocial indicators, the apparent association between the respective factor of interest and the outcome can be flawed. There is conflicting data, whether therapeutic approaches targeting psychosocial factors improve OS.^14^, ^43^, ^44^, ^45^, ^46^, ^47^, ^48^, ^49^ Most studies were conducted in small cohorts, addressing only one of several psychosocial factors. Thus, only very strong effects could have been detected. Prospective studies powered similar to maintenance studies (e.g., in breast cancer) need to address this issue. The CSI might help to (1) explain some conflicting study data, (2) minimise the impact of patient selection by providing a feasible and quick tool (assessment takes less than 5 minutes) for the inclusion of psychosocial parameters in the design of medical procedures, (3) identify patients at risk as psychosocial factors influence adherence to screening and compliance with cancer treatment, (4) identify patients in whom informed consent might be impaired due to a depressive episode or low education and (5) allocate limited resources to patients with a specifically high unmet medical need. The CSI can be easily calculated for individual patients to identify relevant aspects that impact survival by assessing and summing up the included risk factors. Thus, the CSI fosters the aims of personalised medicine as it provides a tool to support the implementation of the most appropriate treatment and healthcare strategy for each patient based on their respective biological and environmental characteristics.

### 4.3 Conclusions

To provide optimal cancer care to each patient, healthcare providers need to be more attentive to the tumour macroenvironment. This includes psychological and social factors; most importantly depressive disorders, previous psychiatric conditions and socioeconomic factors like household income, educational level and employment status. We recommend the CSI to be used in routine clinical practice for the identification of cancer patients at risk of shorter survival.

## CONFLICT OF INTEREST

The authors declare no conflict of interest.

## AUTHOR CONTRIBUTIONS

Alexander Gaiger: Conceptualization, methodology, validation, writing ‐ original draft, supervision, and project administration. Simone Lubowitzki: Formal analysis, investigation, data curation, writing ‐ review & editing, and project administration. Katharina Krammer: Formal analysis, investigation, data curation, writing ‐ review & editing, and project administration. Elisabeth L. Zeilinger: Formal analysis, investigation, data curation, writing ‐ review & editing, and project administration. Andras Acel: Formal analysis, investigation, data curation, writing ‐ review & editing. Olivera Cenic: Formal analysis, investigation, data curation, writing ‐ review & editing. Andrea Schrott: Formal analysis, investigation, data curation, writing ‐ review & editing. Matthias Unseld: Formal analysis, investigation, data curation, writing ‐ review & editing. Anahita Paula Rassoulian: Formal analysis, investigation, data curation, writing ‐ review & editing. Cathrin Skrabs: Investigation, writing ‐ review & editing. Peter Valent: Investigation, writing ‐ review & editing. Heinz Gisslinger: Investigation, writing ‐ review & editing. Christine Marosi: Investigation, writing ‐ review & editing. Matthias Preusser: Investigation, writing ‐ review & editing. Gerald Prager: Investigation, writing ‐ review & editing. Gabriela Kornek: Investigation, writing ‐ review & editing. Robert Pirker: Investigation, writing ‐ review & editing. Günther G. Steger: Investigation, writing ‐ review & editing. Rupert Bartsch: Investigation, writing ‐ review & editing. Markus Raderer: Investigation, writing ‐ review & editing. Ingrid Simonitsch‐Klupp: Investigation, writing ‐ review & editing. Renate Thalhammer: Investigation, writing ‐ review & editing. Christoph Zielinski: Conceptualization, methodology, writing ‐ original draft, and supervision. Ulrich Jäger: Conceptualization, methodology, validation, writing – original draft, and supervision.

## ETHICS APPROVAL STATEMENT

The study was approved by the institutional ethics committee of the Medical University of Vienna, Austria (EC Nr: 2255/2016; 1241/2021).

## PATIENT CONSENT STATEMENT

Informed consent was obtained from each study participant.

## Supporting information


Figure S1
Figure S2

Table S1‐S2

## Data Availability

The data that support the findings of this study are available from the corresponding author upon reasonable request.
