# Supplementary material for: The cancer survival index—A prognostic score integrating psychosocial and biological factors in patients diagnosed with cancer or haematologic malignancies
Source: Cancer Med. 2022 Mar 22;11(18):3387–96. doi: 10.1002/cam4.4697 (PMC9487871; doi:10.1002/cam4.4697)
Supplement: Supplementary file 1 — Figure S1 Figure S2 [file CAM4-11-3387-s001.docx]

**Supplementary Figure S1.** Patients with lower income less frequently articulated need for psychosocial support when depressed than cancer patients with higher income.


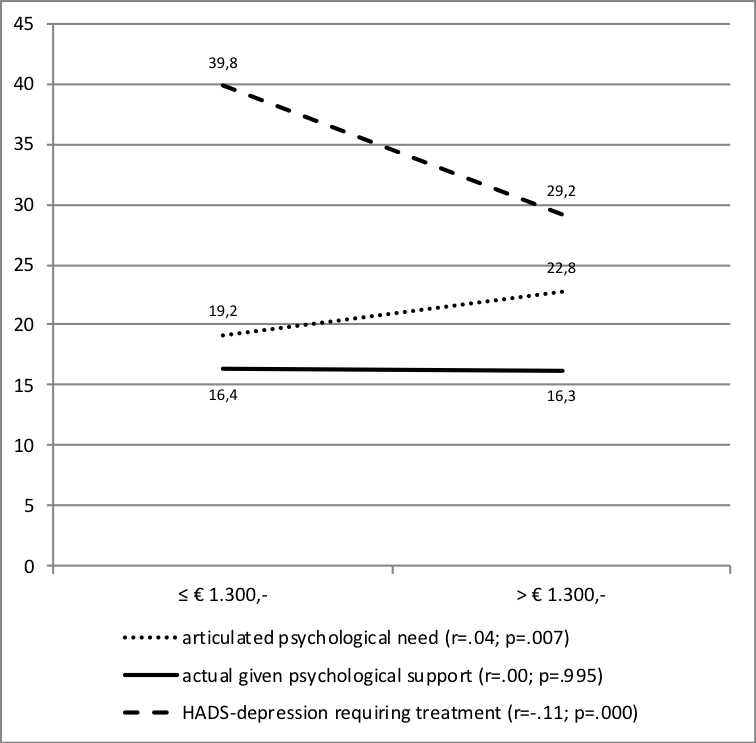


Legend: x-axis, monthly family net income in Euro; y-axis in %

**Supplementary Figure S2.** Patients with lower education less frequently articulated need for psychosocial support when depressed than cancer patients with higher education.


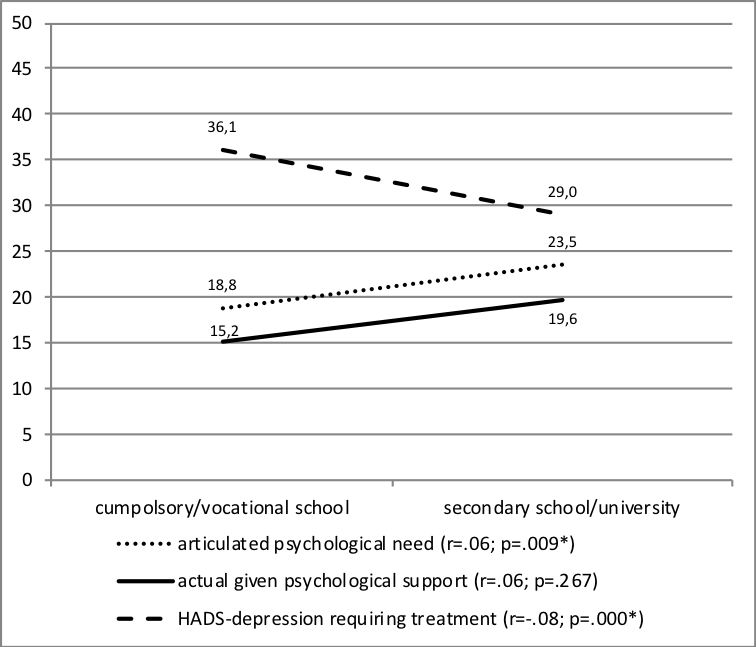


Legend: x-axis, patients education level; y-axis in %
