# Supplementary material for: The cancer survival index—A prognostic score integrating psychosocial and biological factors in patients diagnosed with cancer or haematologic malignancies
Source: Cancer Med. 2022 Mar 22;11(18):3387–96. doi: 10.1002/cam4.4697 (PMC9487871; doi:10.1002/cam4.4697)
Supplement: Supplementary file 2 — Table S1‐S2 [file CAM4-11-3387-s002.docx]

Supplementary Table S1. Patient characteristics

| **Cancer site** | | **Patients** | **Age** | | | **Sex** | **Prevalence of HADS (%)** | | | **Metastatic disease** | **Hb >11** | **Previous psychiatric disorders** | **Unem­ploy­­ment** | **Low income** | **Low edu­cation** |
| --- | --- | --- | --- | --- | --- | --- | --- | --- | --- | --- | --- | --- | --- | --- | --- |
|  | | **N** | **Mean** | **SD** | **Range** | **% male** | **Depression (cut off ≥7)** | **Anxiety (cut-off ≥9)** | **Distress** | **%** | **%** | **%** | **%** | **%** | **%** |
| Total sample | | 2,263 | 58.1 | 13.8 | [18;92] | 48.4 | 32.9 | 24.3 | 29.3 | 29.2 | 77.6 | 9.2 | 4.0 | 37.7 | 53.1 |
| Breast | | 414 | 55.7 | 11.8 | [28;92] | 1.4 | 30.9 | 27.3 | 30.2 | 28.3 | 89.0 | 12.6 | 4.6 | 39.2 | 45.5 |
| Lung | | 241 | 60.4 | 10.2 | [22;83] | 58.5 | 44.8 | 30.3 | 40.2 | 54.4 | 81.4 | 7.5 | 6.4 | 41.7 | 65.5 |
| Colon/rectum | | 146 | 59.8 | 10.9 | [24;83] | 61.0 | 26.7 | 19.2 | 23.3 | 74.0 | 75.9 | 5.5 | 2.8 | 32.0 | 54.8 |
| Lymphoma | | 503 | 58.0 | 15.5 | [18;88] | 54.7 | 29.8 | 21.1 | 26.0 | 0.0 | 80.2 | 9.7 | 3.4 | 35.4 | 50.7 |
| Other cancer sites | | 959 | 58.3 | 14.6 | [18;88] | 39.4 | 33.6 | 24.2 | 29.3 | 32.1 | 70.6 | 8.7 | 3.7 | 3.1 | 54.2 |
|  | Multiple myeloma | 97 | 62.3 | 11.3 | [35;88] | 49.5 | 32.0 | 15.5 | 26.8 | 0.0 | 67.4 | 4.1 | 0.0 | 43.9 | 54.7 |
|  | Acute leukaemia | 76 | 57.9 | 13.6 | [40;82] | 84.6 | 7.7 | 0.0 | 0.0 | 0.0 | 84.6 | 7.7 | 0.0 | 33.3 | 61.5 |
|  | CML | 23 | 63.9 | 14.3 | [32;88] | 34.8 | 21.7 | 17.4 | 17.4 | 0.0 | 47.8 | 0.0 | 0.0 | 36.4 | 52.2 |
|  | HCL | 13 | 57.9 | 10.8 | [40;82] | 84.6 | 7.7 | 0.0 | 0.0 | 0.0 | 84.6 | 0.0 | 0.0 | 33.3 | 61.5 |
|  | Head and neck | 93 | 57.6 | 11.4 | [26;83] | 66.7 | 32.3 | 19.4 | 2.0 | 29.0 | 80.2 | 6.5 | 10.8 | 46.3 | 61.1 |
|  | Oesophagus | 39 | 60.1 | 9.0 | [44;79] | 76.9 | 38.5 | 28.2 | 28.2 | 46.2 | 60.5 | 2.6 | 5.1 | 34.4 | 56.4 |
|  | Stomach | 35 | 61.0 | 13.4 | [30;84] | 77.1 | 37.1 | 34.3 | 37.1 | 65.7 | 62.5 | 14.3 | 0.0 | 45.2 | 59.3 |
|  | Liver and biliary tract | 34 | 62.3 | 10.9 | [28;78] | 76.5 | 29.4 | 23.5 | 23.5 | 64.7 | 67.6 | 11.8 | 2.9 | 52.9 | 50.0 |
|  | Pancreas | 66 | 62.8 | 10.2 | [39;81] | 50.0 | 39.4 | 25.8 | 31.8 | 57.6 | 70.3 | 9.1 | 1.6 | 27.6 | 57.8 |
|  | Sarcomas | 78 | 48.1 | 16.9 | [18;83] | 42.3 | 28.2 | 35.9 | 34.6 | 33.3 | 75.4 | 14.1 | 9.5 | 30.8 | 44.0 |
|  | Gynaecologic | 26 | 56.9 | 12.8 | [31;79] | 0.0 | 50.0 | 46.2 | 32.7 | 53.8 | 62.5 | 23.1 | 3.8 | 61.9 | 50.0 |
|  | Testis | 45 | 37.9 | 12.6 | [18;76] | 100.0 | 15.6 | 20.0 | 15.6 | 20.0 | 94.9 | 6.7 | 2.2 | 23.1 | 40.0 |
|  | Prostate | 56 | 68.7 | 9.7 | [36;88] | 100.0 | 46.4 | 19.6 | 37.5 | 44.6 | 59.1 | 8.9 | 0.0 | 32.7 | 54.5 |
|  | Kidney/renal/pelvis | 40 | 63.8 | 12.0 | [32;81] | 67.5 | 37.5 | 20.0 | 32.5 | 75.0 | 75.0 | 7.5 | 0.0 | 44.7 | 57.5 |
|  | Brain and other CNS | 58 | 49.2 | 13.2 | [23;78] | 51.7 | 37.9 | 32.8 | 32.8 | 0.0 | 92.5 | 15.5 | 5.3 | 36.5 | 50.9 |
|  | Other cancer sites | 180 | 59.0 | 13.6 | [23;72] | 59.5 | 27.0 | 24.3 | 27.0 | 35.1 | 79.3 | 8.1 | 0.0 | 35.3 | 37.1 |

*Legend:* CML, chronic myeloid leukaemia; HCL, hairy cell leukaemia; CNS, central nervous system
